# Resetting of the Human Circadian Melatonin Rhythm by Ambient Hypoxia

**DOI:** 10.1111/jpi.70029

**Published:** 2025-01-17

**Authors:** Titiaan E. Post, Riccardo De Gioannis, Jan Schmitz, Martin Wittkowski, Tina Martin Schäper, Anna Wrobeln, Joachim Fandrey, Marie‐Therese Schmitz, Joseph S. Takahashi, Jens Jordan, Eva‐Maria Elmenhorst, Daniel Aeschbach

**Affiliations:** ^1^ Institute of Aerospace Medicine German Aerospace Center (DLR) Cologne Germany; ^2^ Centre for Human Drug Research (CHDR) Leiden The Netherlands; ^3^ Department of Internal Medicine III—Cardiology University Hospital Cologne Cologne Germany; ^4^ Department of Anesthesiology and Intensive Care Medicine University Hospital Cologne Cologne Germany; ^5^ Institute of Physiology, University Hospital Essen University of Duisburg‐Essen Essen Germany; ^6^ Institute of Medical Biometry, Informatics and Epidemiology, University Hospital Bonn Bonn Germany; ^7^ Department of Neuroscience Peter O'Donnell Jr Brain Institute University of Texas Southwestern Medical Center Dallas Texas USA; ^8^ Howard Hughes Medical Institute University of Texas Southwestern Medical Center Dallas Texas USA; ^9^ Medical Faculty University of Cologne Cologne Germany; ^10^ Institute for Occupational and Social Medicine, Medical Faculty RWTH Aachen University Aachen Germany; ^11^ Institute of Experimental Epileptology and Cognition Research University of Bonn Medical Center Bonn Germany

**Keywords:** circadian clocks, humans, hypoxia, hypoxia‐inducible factors, melatonin, oxygen, phase shifting

## Abstract

Circadian clocks in the body drive daily cycles in physiology and behavior. A master clock in the brain maintains synchrony with the environmental day–night cycle and uses internal signals to keep clocks in other tissues aligned. Work in cell cultures uncovered cyclic changes in tissue oxygenation that may serve to reset and synchronize circadian clocks. Here we show in healthy humans, following a randomized controlled single‐blind counterbalanced crossover study design, that one‐time exposure to moderate ambient hypoxia (FiO_2_ ~15%, normobaric) for ~6.5 h during the early night advances the dim‐light onset of melatonin secretion by 9 min (95% CI: 1–16 min). Exposure to moderate hypoxia may thus be strong enough to entrain circadian clocks to a 24‐h cycle in the absence of other entraining cues. Together, the results provide direct evidence for an interaction between the body's hypoxia‐sensing pathway and circadian clocks. The finding offers a mechanism through which behaviors that change tissue oxygenation (e.g., exercise and fasting/eating) can affect circadian timing and through which hypoxia‐related diseases (e.g., obstructive sleep apnea and chronic obstructive pulmonary disease) can result in circadian misalignment and associated pathologies.

**Trial Registration:** Registration number: DRKS00023387; German Clinical Trials Register: http://www.drks.de

## 1 Introduction

The circadian timing system drives daily cycles in physiology and behavior of many organisms. In mammals, this system consists of a central “clock” located in the suprachiasmatic nucleus (SCN) of the hypothalamus and cell‐autonomous oscillators in most other tissues, referred to as peripheral clocks [1, 2]. The central circadian clock uses light as main entraining cue to maintain synchrony with the environmental day–night cycle and internal signals to uphold phase coherence of peripheral clocks with itself. Misalignment between the central circadian clock and daily behaviors like sleeping and eating—such as occurring in shift work—has been linked to detrimental effects on health and performance, including cardiometabolic disease, sleep disruption, depression, cognitive impairment, and accidents [3, 4, 5, 6, 7]. Such misalignment may also cause *internal* circadian misalignment, an adverse condition in which some peripheral clocks remain synchronized to the central clock whereas others entrain to competing time cues, for example, the fasting/eating cycle [7]. Insight into the nature of internal resetting pathways is thus of great importance both clinically as well as from a basic circadian science perspective.

Several internal factors have been implicated in resetting peripheral clocks, including temperature, glucocorticoids, a cell's redox state, and tissue oxygenation, but whether or not these factors constitute a universal entraining mechanism is not yet clear [8, 9, 10]. Emerging evidence indicates extensive molecular and functional interactions between oxygen‐sensing and circadian pathways [11]. Measurements in rodents revealed daily rhythms in blood and tissue oxygenation, and oxygen cycles within the physiological range were shown to synchronize circadian clocks in cell cultures in a hypoxia‐inducible factor‐1α (HIF‐1α)‐dependent manner. Furthermore, one‐time exposure to moderate hypoxia was reported to accelerate adaptation of rodents' rest–activity rhythm following an advance of the light–dark cycle, suggesting that responsiveness to hypoxia is not limited to peripheral circadian clocks [12].

It has long been suggested that the human circadian system may be responsive to hypoxia [13]. Although changes in the melatonin and core body temperature rhythms during and after exposure to hypoxia have been reported, phase shifting of human circadian clocks under controlled conditions, however, has not been demonstrated [14, 15, 16]. Here, we present results from a study in healthy humans in which we compared the circadian timing of melatonin secretion before and after exposure to moderate hypoxia and normoxia (control) under strictly controlled conditions (continuous dim light, constant posture [CP], and time‐free environment). Given the suspected role of the transcription factor HIF‐1 in circadian phase resetting [11], we explored in the blood whether exposure to moderate hypoxia activates transcription of *HIF‐1A* and/or several target genes of the HIF‐1 protein.

## 2 Materials and Methods

The protocol was approved by the ethics board of the North Rhine Medical Association (Ärztekammer Nordrhein; protocol number: 2020075) and registered at the German Clinical Trials Register (www.drks.de, registration number: DRKS00023387). Informed written consent was obtained from study participants before the start of the study. All procedures were conducted according to the Declaration of Helsinki (2013). Study participants were compensated for their participation. The study was carried out in the *envihab* research facility (www.dlr.de/envihab/) of the German Aerospace Center (DLR) in Cologne, Germany, which features private rooms equipped for sleep recordings, around‐the‐clock blood sampling, and various atmospheric conditions including normobaric hypoxia.

### 2.1 Study Participants and Screening Procedures

Between September 2020 and July 2022, 198 interested volunteers entered a multistep screening procedure, resulting in the selection of 22 healthy adults (12 females; mean age ± SD: 25.18 ± 2.68 years; range: 20–30 years). Study participants were of intermediate chronotype (Munich ChronoType Questionnaire, MCTQ; MSFsc = 3:00–5:00 h) and were in good health as established by Pittsburgh Sleep Quality Index, PSQI < 8, medical history, physical examination, routine blood and urine testing, and overnight polysomnography (apnea–hypopnea index, AHI < 10; periodic leg movements in sleep, PLMS < 15). Participants reported no night shifts for 3 months before the study and no air travel across multiple time zones within the past month. Participants were asked to abstain from use of drugs, caffeine, alcohol, and nicotine 1 week before each admission to the laboratory, which was verified by urine testing upon admission.

### 2.2 Study Protocol

**Figure 1:**
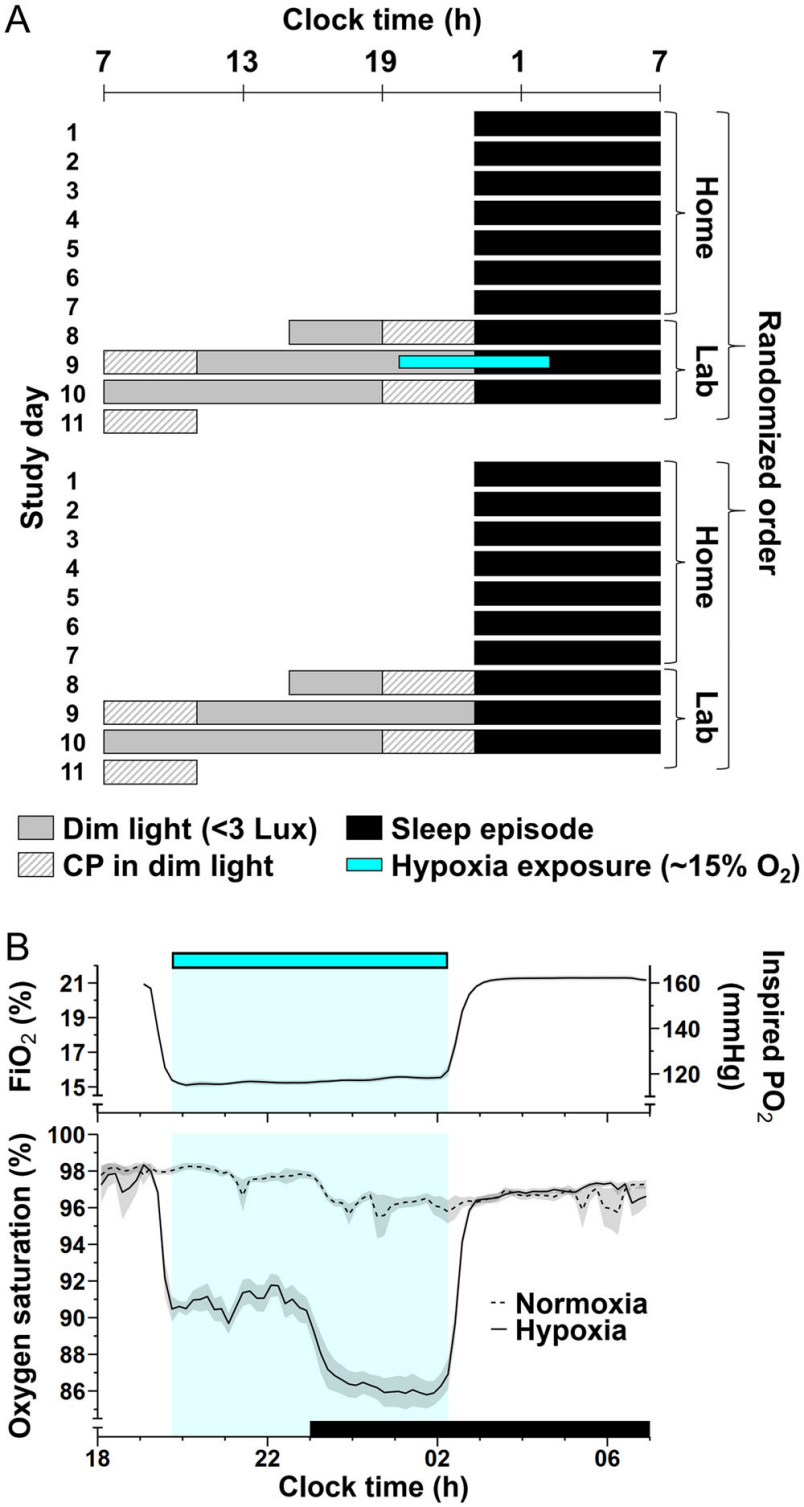
Representation of the study design, atmospheric conditions, and participants' blood oxygenation. (A) Raster plot of the study protocol. The protocol consisted of two conditions that were 11 days long and each included a 4‐day laboratory visit during which participants were exposed either to normobaric hypoxia or to normoxia (control). Black bars indicate the 23:00–07:00 h scheduled sleep episodes. in darkness. Gray bars denote dim room light (< 3 lux in the angle of gaze; see Section 2.2). Striped bars show the constant posture (CP) procedures during wakefulness in dim room light. The blue bar denotes the scheduled hypoxia exposure (19:45–02:15 h). Participants were randomized to the order of atmospheric condition (crossover, counterbalanced, and single‐blind). (B) Atmospheric conditioning and blood oxygen saturation levels during hypoxia exposure and control. (*Upper panel*) Fraction of inspired oxygen and corresponding oxygen partial pressure during 6.5 h of normobaric hypoxia exposure (blue bar and shade). (*Lower panel*) Mean (± SEM) blood oxygen saturation (SpO_2_) profiles during hypoxia exposure and control. The black bar denotes the scheduled sleep episode. *N* = 14 due to accidental late start of the SpO_2_ recordings in eight participants.

We used a single‐blind counterbalanced crossover design with two conditions, each of which was 11 days in duration and included a 4‐day laboratory visit (Figure 1A and Supporting Information S1: Figure S1), scheduled in block‐randomized order (blocks of 6–8). In the active condition, participants were exposed to normobaric hypoxia (FiO_2_ = ~15%) for ∼6.5 h centered at 23:00 h on Day 9, whereas no such exposure occurred in the control condition (normobaric normoxia, FiO_2_ = 21%). During the first 7 days at home as well as during the laboratory visit of each condition, participants followed a fixed sleep schedule (23:00–7:00 h). Before the laboratory visits, wrist actigraphy (Actiwatch‐L; Philips/Respironics) and daily call‐ins into a voicemail system were used to check compliance with the sleep instructions. The 4‐day laboratory visits were 10–11 days apart. During the visits, participants remained in private rooms in dim light during scheduled wakefulness and in complete darkness during scheduled sleep episodes (Figure 1A). LED‐based ceiling lights covered with dark window film were the only light source in the rooms (< 3 lux, < 0.006 W/m^2^, < 0.9 melanopic IDE [17] in the angle of gaze, color temperature 2900–2990 K; measurements obtained at study start [spectrometer: BLACK‐Comet‐CXR‐SR‐50, StellarNet Inc.], illuminance checked again at study end). Room temperature was maintained at ~22.2°C. Participants were monitored by cameras inside their rooms to allow staff to enter and if needed prevent them from falling asleep outside the scheduled sleep episodes. Participants strictly adhered to meal times at 8:00, 12:00, 16:30, and 20:30 h (snack) and had 30 min to consume the meals. The meals were of mixed content; the macronutrient content and caloric intake were not controlled. Participants had no access to television, video, and electronic devices and remained uninformed about the time of day.

### 2.3 Atmospheric Conditions

Hypoxia was achieved by nitrogen dilution through the air handling system in the atmospheric self‐sustained hypoxia chamber under normobaric conditions (~760 mmHg). Nitrogen was supplied by an external tank, resulting in an oxygen fraction of ~15% and an oxygen partial pressure (PO_2_) of ~115 mmHg. Oxygen availability thus approximated the maximum cabin altitude (8000 ft or 2438 m) as allowed during normal operation of an airliner. The target oxygen concentration was reached 30 min after the start of nitrogen dilution (Figure 1B). We measured blood oxygenation (SpO_2_) levels continuously using a finger pulse oximeter (OEM Oximetry module, Nonin), starting on Day 9 at 18:00 h (*N* = 14) or 23:00 h (unintended late start of recording, *N* = 8) and ending on Day 10 at 7:00 h.

**Figure 2:**
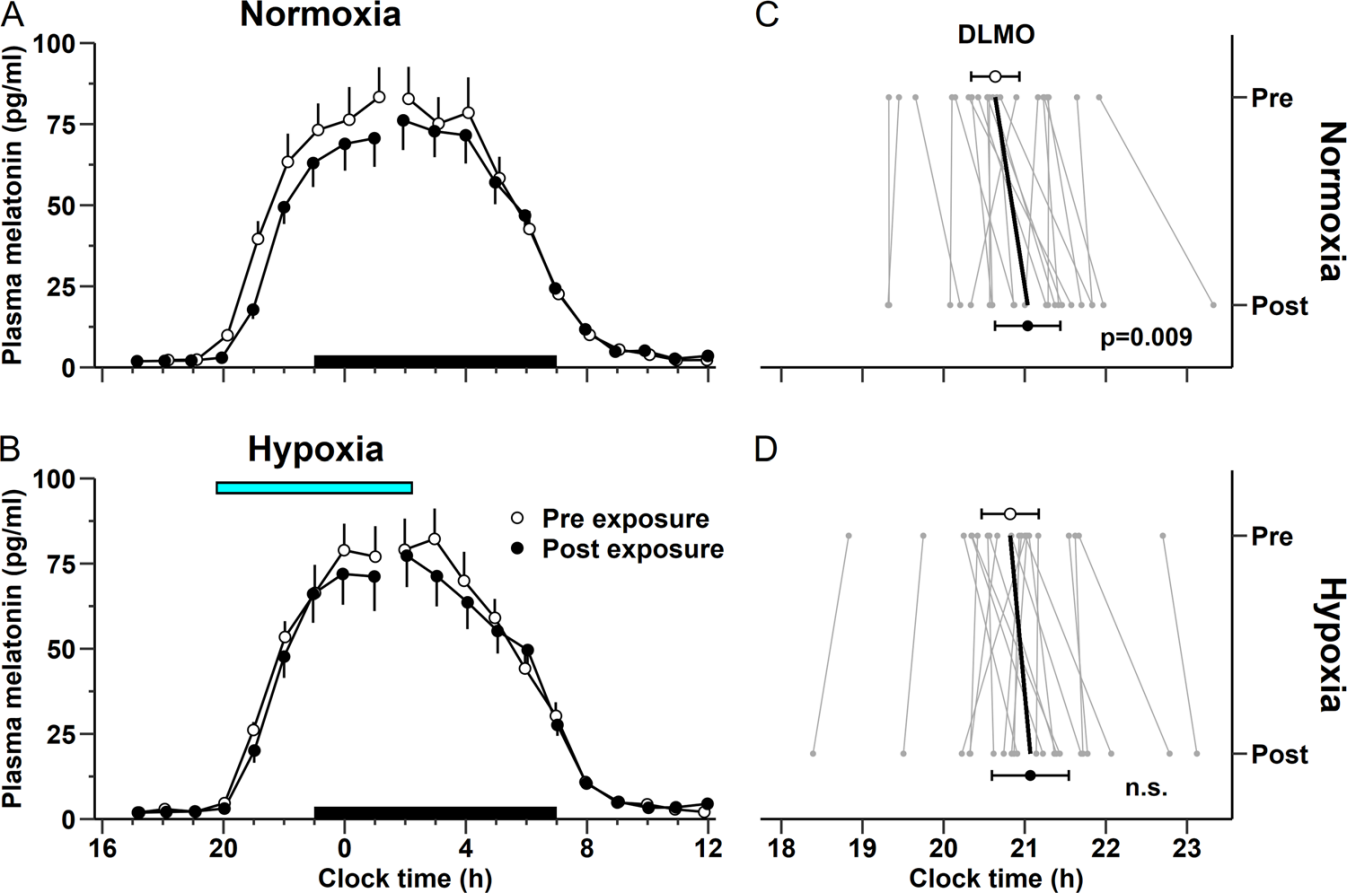
Average plasma melatonin profiles and individual dim light melatonin onset (DLMO) times during nights before and after hypoxia exposure and control. Data in (A) and (B) show mean (± SEM) plasma melatonin levels in the control (*n* = 22) and hypoxia (*n* = 21) condition, respectively. Time series were split and aligned relative to mean times of DLMO_25%_ and dim light melatonin offset (DLMOff_25%_). The black bars indicates the scheduled sleep episodes. The blue bar denotes the scheduled hypoxia exposure (19:45–02:15 h) during the night in between melatonin measurements. Data in (C) and (D) show individual time points of the DLMO_25%_ and group averages (95% CI) in the two conditions. *p* Value indicates significant drift of DLMO_25%_ between pre‐ and post‐normoxia exposure (*t* test).

### 2.4 CP Procedures

CP procedures occurred before (beginning on Day 8) and after (beginning on Day 10) the treatment night of each visit, for the assessment of circadian phase of the melatonin rhythm (Figure 1A). Participants remained for a 16‐h period in bed in a semi‐recumbent posture with minimal activity for 4 h before and 4 h after the 8‐h sleep episode and were required to sleep in the same position. Room temperature and dim light conditions remained constant during the CP except for the sleep episodes that took place in complete darkness.

### 2.5 Plasma Melatonin

Hourly blood samples were collected during CPs via an indwelling forearm intravenous catheter before and after treatment nights for measurement of plasma melatonin levels. During the sleep episode, samples were taken from outside the rooms through a port hole without disturbing the participants' sleep. Samples were collected and then frozen (−80°C) for subsequent assay. Plasma melatonin samples were assayed (Chrono@Work, Groningen, the Netherlands) using liquid chromatography in combination with mass spectrometry (LCMS/MS), which had a functional sensitivity of 2.3 pg/mL and an analytical sensitivity of 1.9 pg/mL, an intraassay precision of 3.5%–8.9% for low to high concentrations, and interassay precision of 4.1%–9.5%.

### 2.6 Circadian Phase Assessment

The dim light melatonin onset (DLMO) served as the marker of central clock timing. Circadian phase of DLMO_25%_ was calculated as the time at which levels of melatonin rose above 25% of the peak‐to‐trough amplitude during the 17‐h sampling interval: DLMO_25%_ = (melatonin amplitude − daytime melatonin level) × 25/100 + daytime melatonin level [18]. The melatonin amplitude was calculated as the difference between the maximum and the daytime melatonin levels. To avoid inflation by a local maximum, the median of the three highest melatonin values during the 17‐h interval was chosen as the maximum melatonin value. To determine the daytime melatonin level, the median of the three lowest melatonin values was chosen. A linear interpolation between the melatonin values just below and just above the 25% threshold was used to identify the time of DLMO_25%_. First, phase shifts were calculated as the difference in the time of DLMO_25%_ before (pre) and after (post) treatment (hypoxia or control). A potential phase shift due to hypoxia exposure (primary outcome) was assessed by subtracting in each individual the phase shift in the control condition (corresponding to the circadian drift) from the phase shift in the hypoxia condition. One participant was excluded from analysis of DLMO timing due to an inability to draw blood during a period of one of the CP procedures. Therefore, hypoxia‐induced phase shifting was determined in 21 participants.

In addition to using DLMO_25%_, we examined a potential phase‐shifting effect of hypoxia on the basis of DLMO_10_, that is the time at which plasma melatonin crosses the absolute threshold of 10 pg/mL [19, 20].

### 2.7 RNA Isolation and Real‐Time PCR of HIF, HIF Target Genes, and Clock Genes

Transcription levels of HIF‐1α and HIF‐2α (*HIF1A* and *HIF2A*), HIF target genes (glucose transporter type 1 [*SLC2A1*], vascular endothelial growth factor [*VEGF*], adrenomedullin [*ADM*], pyruvate dehydrogenase kinase 1 [*PDK1*], prolyl hydroxylases 1, 2, and 3 [*PHD1, PHD2*, and *PHD3*]), and clock genes (aryl hydrocarbon receptor nuclear translocator‐like [*ARNTL*], circadian locomotor output cycles kaput [*CLOCK*], cryptochrome circadian regulator 1 and 2 [*CRY1* and *CRY2*], albumin gene d‐site‐binding protein [*DBP*], nuclear receptor subfamily 1, group D, member 1 [*NR1D1*], period circadian regulator 1, 2, and 3 [*PER1, PER2*, and *PER3*], retinoic acid‐related orphan receptor A [*RORA*]) were assessed once before and twice during exposure to hypoxia and normoxia (i.e., 3.5 and 6.25 h after beginning the treatment). Total RNA from whole blood was directly stabilized using PAXgene Blood RNA Tubes (PreAnalytiX GmbH) and then frozen (−20°C). RNA was isolated according to the PAXgene Blood RNA Kit (PreAnalytiX GmbH) and reverse‐transcribed using M‐MLV reverse Transcriptase (Promega GmbH, Walldorf, Germany). Real‐time polymerase chain reaction (RT‐PCR) was performed with the Biozym Blue S'Green qPCR‐Kit (Biozym Scientific GmbH, Oldendorf, Germany) on a Bio‐Rad's CFX96 real‐time system (Bio‐Rad Laboratories GmbH, Feldkirchen, Germany). We reverse‐transcribed 200 ng of total RNA into cDNA, which was amplified with gene‐specific primers (Supporting Information S1: Table S1) and normalized to *ACTB* (β‐actin). Primer specificity was checked by Primer‐BLAST and confirmed by size analysis of the PCR amplicons [21]. Expression was calculated with the 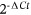 method for statistical analysis. A list of the primer sequences used for qRT‐PCR analysis is provided in Supporting Information S1: Table.

### 2.8 Statistical Analysis

We hypothesized that the pre–post difference in DLMO timing (primary outcome) would differ between hypoxia and control. Accordingly, sample size was estimated for the primary outcome. Given the absence of previous data, a two‐stage approach was employed. After stage one, an interim analysis of DLMO_25%_ was conducted with the data from the first seven participants (three females; one additional participant was excluded due to incomplete data during one of the CPs). Conditional power considerations based on a two‐sided *t* test with an *α* of 0.05 and a mean of the DLMO difference (hypoxia‐normoxia) of 8.2 min with SD of 13.9 min achieved a power above 80% for a total of 21 participants. The second stage of the study involved an additional 14 participants (eight females). The overall results of both stages, before and after the interim analysis, were combined using the inverse normal *p*‐value combination method [22]. The study was not intended to or had any specific hypotheses to test for potential effects of sex on the primary outcome and thus was not powered accordingly.

The RT‐PCR sets were analyzed for Gaussian distribution using the D'Agostino–Pearson, Anderson–Darling, and Shapiro–Wilk test. Gaussian‐distributed data were analyzed via mixed‐effects analysis for repeated measurements. Non‐Gaussian distributed data sets were either transformed to Gaussian distributed data and analyzed accordingly or analyzed via the Friedman test for repeated measurements. Statistical analyses were performed using the R language and environment for statistical computing (version 2022.07.1) and GraphPad Prism (version 8.0.2) from GraphPad Software Inc. *p* Values < 0.05 were considered statistically significant [23].

## 3 Results

### 3.1 Ambient Hypoxia Lowers Blood Oxygen Saturation, Particularly During Sleep

Exposure to normobaric hypoxia (Figure 1B) lowered SpO_2_ during the interval of scheduled wakefulness (19:45–23:00 h) to an average of 90.9% (95% CI: 90.6%–91.1%) and during the interval that overlapped with the sleep episode (23:00–02:15 h) to 86.5% (86.1%–86.9%). In total, participants spent 247 min (231–263 min) below 90% SpO_2_ and 49 min (38–60 min) below 85%. Exposure to normoxia resulted in an average SpO_2_ of 97.8% (97.7%–97.9%) during scheduled wakefulness and 96.3% (96.1%–96.5%) during the interval that overlapped with the sleep episode. In total, participants spent 5 min (1–8 min) below 90% SpO_2_ and 2 min (0–4 min) below 85%.

### 3.2 Early‐Night Exposure to Hypoxia Advances the Timing of DLMO

**Figure 3:**
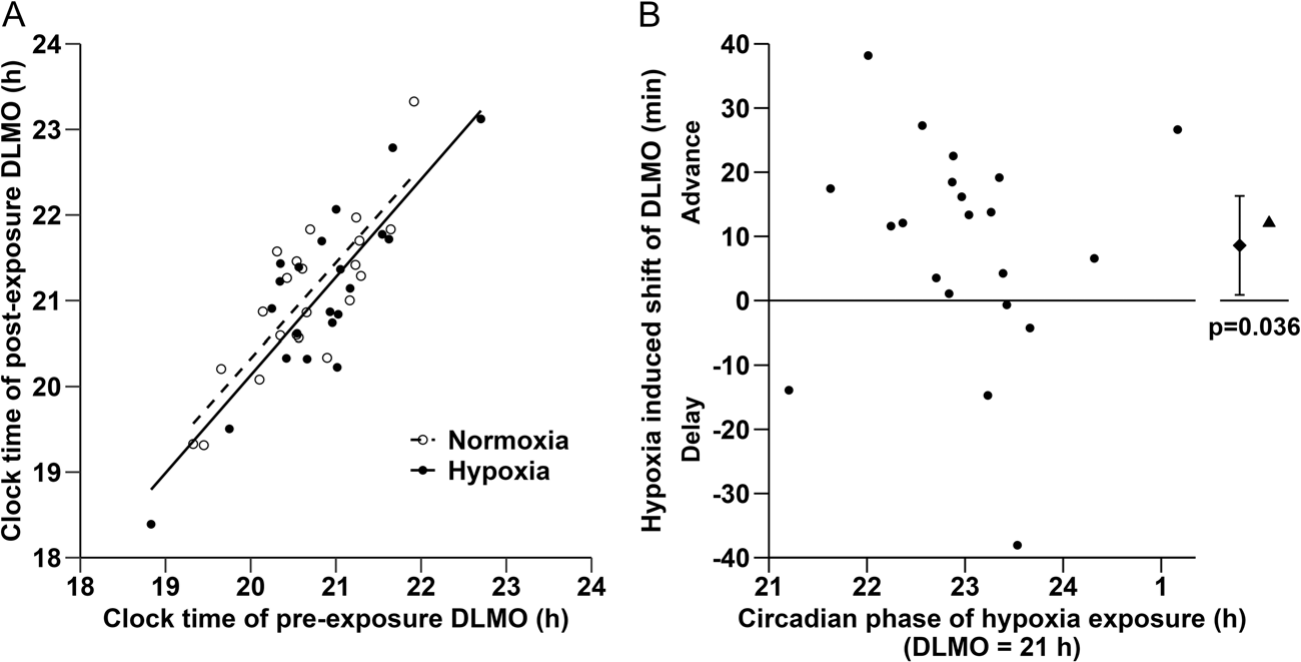
Resetting of individual dim light melatonin onset (DLMO) by ambient hypoxia. (A) Post‐exposure DLMO_25%_ times are plotted against pre‐exposure DLMO_25%_ times for the hypoxia and control condition (*N* = 21). A decrease of the *y*‐intercept of the regression line illustrates an overall advance of DLMO after hypoxia exposure compared to control. (B) Individual phase shifts due to hypoxia (filled dots) are plotted against the circadian phase of the midpoint of the hypoxia interval, with 21 h defined as the time of estimated DLMO during the exposure night. Phase shifts were corrected for each individual's circadian drift during dim light control in normoxia (see Figure 2C). Mean phase shift with 95% CI (filled diamond) and median (filled triangle) are shown on the right (*N* = 21); *p* value indicates significant difference from 0 (*t* test).

The time course of mean plasma melatonin levels on the days before and after hypoxia exposure and on the corresponding days in the control condition (normoxia) are illustrated in Figure 2. In the control condition, the timing of the DLMO_25%_ was delayed on average by 24 min (9–38 min, *p* = 0.009) between pre‐ and postexposure (Figure 2C), reflecting the intrinsic period–dependent phase drift in dim light. In contrast, no significant delay of DLMO_25%_ was observed after hypoxia exposure (15 min, 0–30 min, *p* = 0.062, Figure 2D). Linear regression of individual postexposure DLMO_25%_ time with preexposure DLMO_25%_ time indicated a phase advance due to hypoxia as illustrated by a decrease in the *y*‐intercept in the hypoxia condition compared to control (Figure 3A). To correct for each individual's circadian phase drift in dim light, the pre–post difference in the timing of DLMO_25%_ in the control condition was subtracted from the pre–post difference in the timing of DLMO_25%_ in the hypoxia condition. The result revealed an average phase advance of 9 min (1–16 min, *p* = 0.036) due to hypoxia exposure (Figure 3B). Sixteen out of 21 participants (76%) showed a phase advance attributed to hypoxia exposure. Similar results were obtained for DLMO_10_: in this case the mean hypoxia‐induced phase advance was 11 min (1–21 min, *p* = 0.037).

The magnitude of phase shifting did not correlate with the individual circadian phase of hypoxia exposure (Figure 3B; Spearman *r* = −0.21, *p* = 0.36). Due to the study design, however, the range of individual phases of exposure was quite narrow (mean difference between estimated DLMO_25%_ and midpoint of hypoxia exposure: 1.98 h, 95% CI: 1.59–2.38 h).

Hypoxia exposure did not induce a significant phase shift of DLMOff_25%_ (4 min, −14–21 min, *p* > 0.4). Moreover, neither the duration of the nocturnal melatonin profile (*p* > 0.3) nor its amplitude (*p* > 0.5) was affected by exposure to hypoxia. Table 1 provides an overview of the timing of DLMO_25%_ and DLMOff_25%_, as well as the duration and amplitude observed during each CP.

**Table 1:** Timing, duration, and amplitude of the plasma melatonin profiles before and after exposure to hypoxia and normoxia.

	**Pre‐normoxia**	**Post‐normoxia**	**Pre‐hypoxia**	**Post‐hypoxia**
DLMO_25%_ (h:min)	20:41	21:05*	20:49	21:04#
	(20:23–20:59)	(20:40–21:29)	(20:28–21:10)	(20:36–21:33)
DLMOff_25%_ (h:min)	07:06	07:16	07:14	07:21
	(06:38–07:33)	(06:48–07:44)	(06:48–07:40)	(06:47–07:55)
Duration (h)	10.41	10.19	10.42	10.28
	(10.25–10.58)	(10.14–10.24)	(10.34–10.50)	(10.19–10.37)
Amplitude (pg/mL)	85.4	76.7	81.1	78.7
	(64.8–106.1)	(58.9–94.5)	(63.5–98.8)	(59.7–97.6)

*Note:* The times for dim light melatonin onset (DLMO_25%_) and dim light melatonin offset (DLMOff_25%_) refer to clock time. Duration is the difference between the times of DLMO_25%_ and DLMOff_25%_. Values are expressed as means (*N* = 21) with 95% CI shown in parentheses. For DLMOff_25%_, duration, and amplitude no pre–post difference, or difference of pre–post difference between hypoxia and normoxia conditions was observed.

* *p* < 0.05 for pre–post difference (*t* test).

# *p* < 0.05 for difference of pre–post difference between hypoxia and normoxia conditions (*t* test).

We explored whether the duration of an individual's hypoxemia (i.e., the total time during which SpO_2_ fell below 90% or below 85%) was a predictor of the magnitude of the hypoxia‐induced phase shift of DLMO_25%_. However, in neither case was there a significant correlation (below 90%: Spearman *r* = −0.20, *p* = 0.37; below 85%: *r* = −0.21, *p* = 0.35).

### 3.3 No Changes in HIF and HIF‐Dependent Gene Transcription in Leukocytes During Hypoxia Exposure

In whole blood, mRNA levels of HIFs (*HIF1A* and *HIF2A*), HIF target genes (*SLC2A1, VEGF, ADM, PDK1, PHD1, PHD2,* and *PHD3*), and clock genes (*ARNTL, CLOCK, CRY1, CRY2, DBP, NR1D1, PER1, PER2, PER3*, and *RORA*) were not different during hypoxia and normoxia compared to the pre‐exposure measurement, or between the two conditions (Supporting Information S1: Figures S2 and S3).

## 4 Discussion

We found that exposure of healthy adults to a 6.5‐h interval of normobaric hypoxia early in the night advanced DLMO_25%_ by 9 min and DLMO_10_ by 11 min. To our knowledge, this is the first controlled study demonstrating that hypoxia may act as a zeitgeber of the central circadian clock in humans. A phase advance of 9 min may seem small but, remarkably, it matches the extent to which the intrinsic circadian period of healthy adults (24 h 9 min; females: 24 h 5 min, males: 24 h 11 min [24]) deviates from 24 h, when measured in an environment with minimal confounding influences. Thus, exposure to moderate hypoxia early in the night may be strong enough to entrain circadian clocks to a 24‐h cycle in the absence of other time cues.

Despite the fact that hypoxia induced a phase advance of DLMO while the duration of the melatonin profile did not differ from the control condition, we did not find a significant advance of DLMOff. This discrepancy can be explained by the larger interindividual variance of DLMOff compared to DLMO due the contribution of other, likely non‐circadian factors, including differences in melatonin's clearance from the blood. In general, DLMO is a more reliable marker of central clock timing than DLMOff [25] and considered the “gold standard” in circadian phase assessment in humans. In contrast to a previous report [15], we did not find a decrease in the melatonin amplitude after hypoxia exposure. The former observation likely represents an acute aftereffect of hypoxia—possibly due to sympathetic activation—and not a circadian effect, given that hypoxia exposure ended only ~6 h before melatonin secretion, whereas in the current study it did so ~22 h before the onset of secretion.

The hypoxia exposure in the current study is considered moderate. However, one should keep in mind that the co‐occurrence with sleep—likely due to the associated change in breathing pattern—resulted in considerable oxygen desaturation similar to previous findings [26], such that participants spent > 4 h below the clinical hypoxia threshold of 90% SpO_2_ and even ~50 min below 85%. Such desaturation is comparable to observations in patients with obstructive sleep apnea (OSA) [27]. This disorder is associated with disrupted respiration, fragmented sleep, and cardiometabolic dysregulation. Internal circadian misalignment due to intermittent hypoxia has been implicated as a contributing factor of OSA sequelae in a recent study [28]. While that study used a mouse model and exposure to severe hypoxia (6%), our data provide direct human evidence that even moderate hypoxemia may interfere with circadian timekeeping. Consistent with this finding, a recent exploratory analysis observed internal misalignment between the circadian rhythms of blood pressure and melatonin in OSA patients [29]. Apart from OSA, our results are relevant for chronic lung diseases, since circadian clock disruption appears to play an important role in those pathologies as well [30].

We propose that changes in tissue oxygenation may serve as a mediator of various non‐photic phase‐shifting stimuli:

1. Exercise is known to shift circadian rhythms in humans [31, 32] and to affect oxygenation of various tissues including the brain. Cerebral oxygenation showed a quadratic response to incremental exercise, increasing with moderate intensities and decreasing at high intensity [33]. In mice, strenuous exercise‐induced clock‐ and HIF‐1α target genes and this response also depended on time of day [34].
2. Eating at night can shift peripheral circadian rhythms and even uncouple them from central clock control [7]. Eating/nutrient processing is associated with changes in tissue‐specific blood flow and oxygen consumption that in addition to the rest–activity rhythm likely contribute to the observed daily rhythms in tissue oxygenation. Conversely, oxygen cycles within the physiological range can synchronize cellular clocks in a HIF‐1α‐dependent manner [12]. It should be noted, however, that recent work in mice also suggested a role for carbon dioxide in feeding‐mediated clock resetting [35].
3. Exposure to high altitude, which is associated with reduced oxygen partial pressure in the air, alters time‐of‐day‐dependent variations in the human blood transcriptome [36]. Moreover, changes in daily physiological rhythms were observed in experiments in an altitude chamber [37]. Remarkably, human chronotype, which has been linked to the length of an individual's intrinsic circadian period [24], was reported to vary between groups living at different altitudes [38]. Thus, it is possible that apart from acute effects, there may be long‐term adaptive/genetic effects of hypoxia exposure on the human circadian system.
4. Trans‐meridian flights cause circadian misalignment, which is associated with sleep disruption, fatigue, mood disturbance, and gastrointestinal symptoms, collectively referred to as jetlag. The present study indicates that the level of hypoxia typically experienced in an airliner at cruising altitude may play a role in jetlag. Accelerated adaptation to an advance of the light–dark cycle—corresponding to an eastbound flight—was reported after hypoxia exposure in a mouse model of jetlag [12].

The hypoxia‐induced phase advance observed in the present study did not appear to be mediated by changes at the level of HIF‐1α gene transcription. The HIF pathway is known to be activated at the protein level through stabilization of HIF‐1α under conditions of reduced oxygen availability [39]. HIF‐1, which is composed of the subunits HIF‐1α and HIF‐1β, regulates the expression of genes including clock genes. Currently, there is no reliable method established to measure HIF‐1α protein in human peripheral leukocytes. Our approach was therefore to quantify transcription of HIF‐1 target genes. Since we did not find hypoxia‐induced changes in gene transcription, we conclude that the oxygen partial pressure in the blood was not low enough to elicit significant HIF‐1 activation in the majority of circulating leukocytes. However, oxygen partial pressure varies considerably across tissues even under normoxia [40]; it has been reported to be lower in brain tissue—particularly, in deeper layers—than in venous blood [41, 42], making activation of HIF‐1 following a decrease in the inspiratory oxygen partial pressure more likely in the former than in the latter. Moreover, in mice, nuclear HIF‐1α accumulation following hypoxia exposure differed between tissues and in some cases was scant, suggesting the involvement of other transcription factors in the response to hypoxia [28].

## 5 Limitations of the Study

To maximize statistical power, we chose to administer hypoxia only at one time of day in all participants. Thus, we were not able to examine possible time‐of‐day–dependent (and ultimately circadian‐phase–dependent) effects of hypoxia. An exploratory analysis in which the magnitude of the hypoxia‐induced phase shift was expressed relative to the time of each individual's estimated DLMO did not reveal a significant relationship—possibly due to the limited interindividual variation in initial DLMO time. It remains to be investigated whether hypoxia exposure can induce phase delays, and ultimately whether its effect on the circadian system can be adequately described by a phase–response curve—similar to established entraining cues like light or melatonin. In the absence of a demonstrated phase delay, it cannot be excluded that hypoxia induces a general shortening of the intrinsic circadian period, similar, for example, to the action of small‐molecule inhibitors of glycogen synthase kinase 3 (GSK‐3) [43]. Notably, inhibition or depletion of GSK‐3 can lead to HIF‐1α induction, while GSK‐3β overexpression reduces HIF‐1α levels [44]. These findings hint at a promising link between the modulation of GSK‐3 activity, the regulation of circadian rhythms, and hypoxia. Finally, whereas our study focused on melatonin as a marker of the central clock, and although central and peripheral clocks appear to share identical core molecular clockworks [2], it needs to be established whether hypoxia exposure can reset peripheral clocks in humans, as reflected, for example, in glucose and insulin rhythms during a constant routine protocol [7].

## Author Contributions

**Titiaan E. Post:** conceptualization, methodology, project administration, investigation, formal analysis, visualization, writing–original draft, writing–review and editing. **Riccardo De Gioannis:** methodology, investigation. **Jan Schmitz:** investigation. **Martin Wittkowski:** methodology, investigation. **Tina Martin Schäper:** methodology, investigation, formal analysis, visualization, writing–review and editing. **Anna Wrobeln:** formal analysis, visualization, writing–review and editing. **Joachim Fandrey:** methodology, writing–review and editing. **Marie‑Therese Schmitz:** formal analysis. **Joseph S. Takahashi:** methodology, writing–review and editing. **Jens Jordan:** conceptualization, writing–review and editing. **Eva‐Maria Elmenhorst:** conceptualization, methodology, investigation, writing–review and editing, supervision. **Daniel Aeschbach:** conceptualization, methodology, visualization, writing–original draft, writing–review and editing, supervision, funding acquisition. All authors approved the final manuscript.

## Ethics Statement

The protocol was approved by the ethics board of the North Rhine Medical Association (Ärztekammer Nordrhein; protocol number: 2020075).

## Conflicts of Interest

J.S.T. is a co‐founder and scientific advisory board member of Synchronicity Pharmaceuticals Inc. The other authors declare no conflicts of interest.

## Supporting information

Supporting information.

## Data Availability

The data that support the findings of this study are openly available in Open Science Framework at https://osf.io/s8yb2/, reference number DOI 10.17605/OSF. IO/S8YB2.
