# Supplementary material for: Resetting of the Human Circadian Melatonin Rhythm by Ambient Hypoxia
Source: J Pineal Res. 2025 Jan 17;77(1):e70029. doi: 10.1111/jpi.70029 (PMC11740168; doi:10.1111/jpi.70029)
Supplement: Supplementary file 1 — Supporting information. [file JPI-77-e70029-s001.pdf]

Supplementary Materials for

Resetting of the human circadian melatonin rhythm by ambient hypoxia

Titiaan E. Post *et al.*

*Corresponding author. Email: daniel.aeschbach@dlr.de

This PDF file includes:

Figs. S1 to S3
Table S1

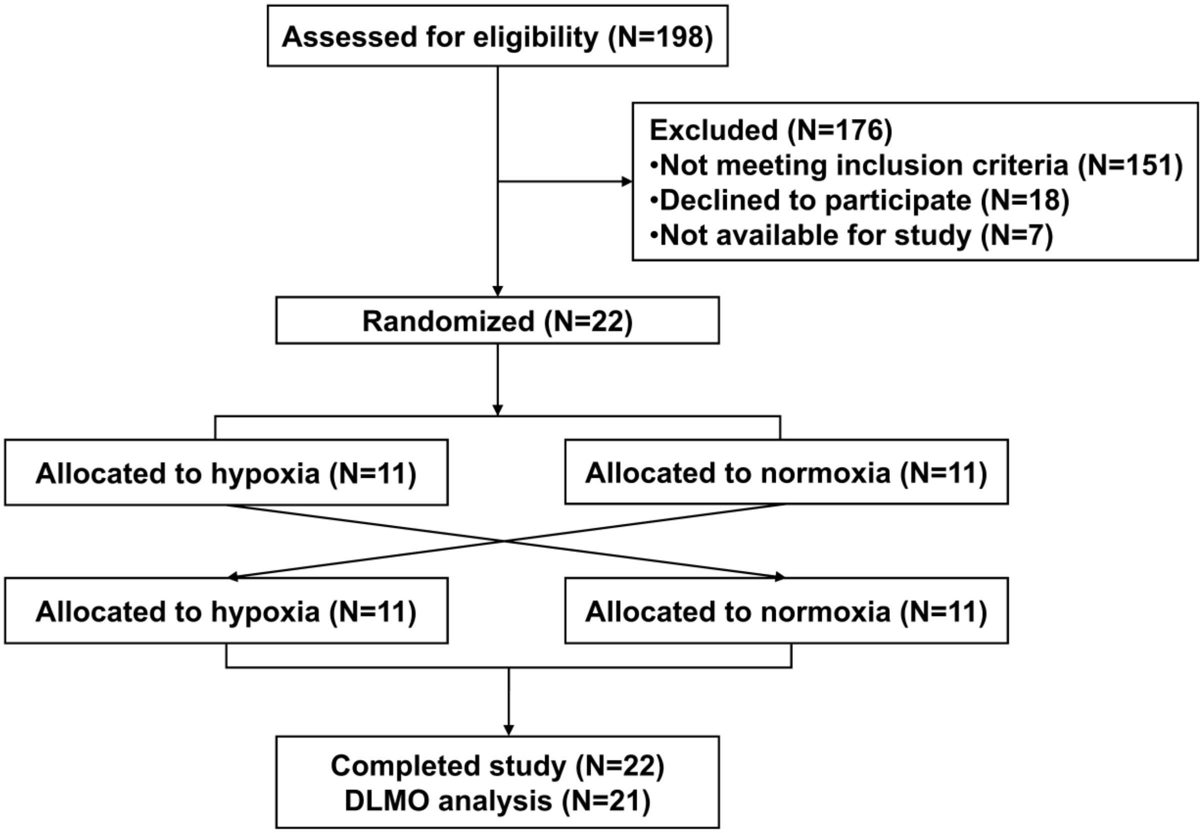

Fig. S1. Flow diagram of the study.

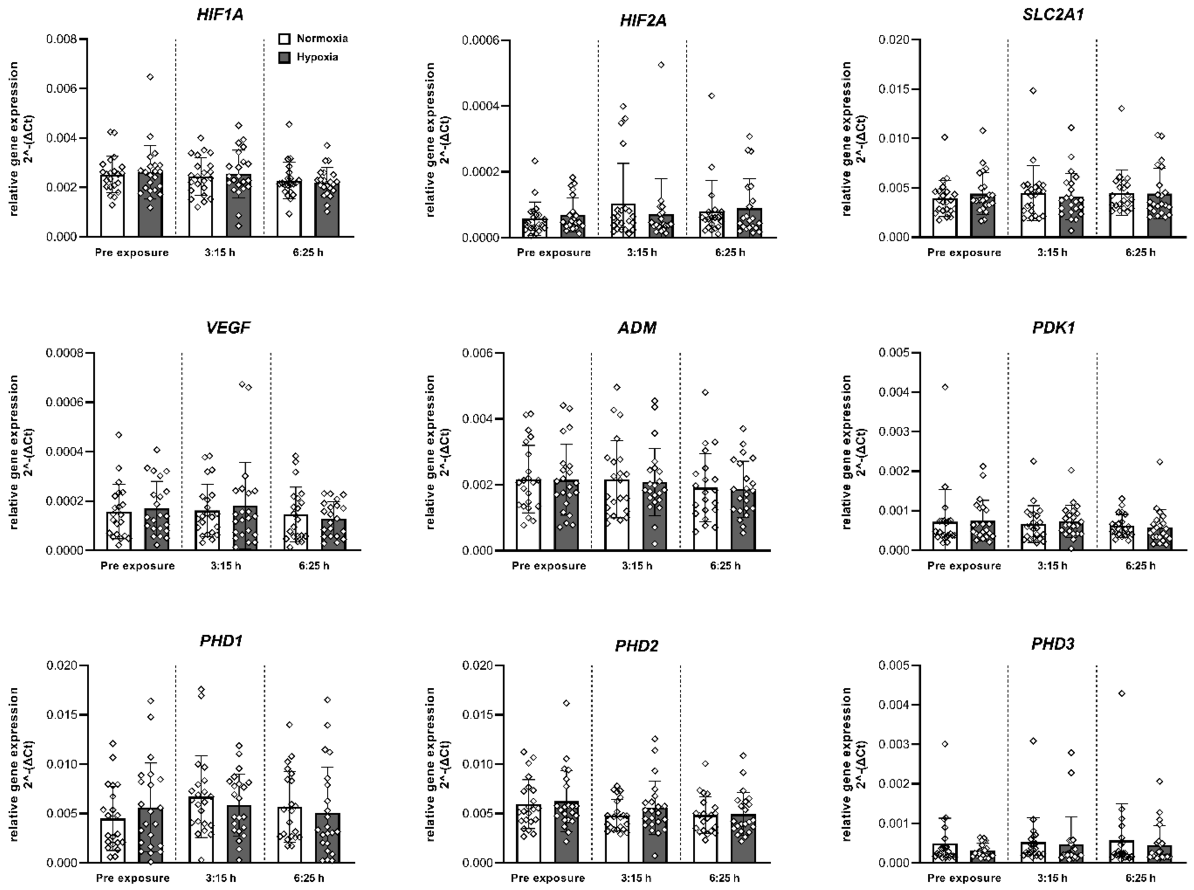

Fig. S2. Expression of *HIF1A*, *HIF2A* and HIF target genes in leukocytes collected from participants before and during exposure to 6.5 hours of normobaric hypoxia (FiO_2=15%) and normoxia (FiO_2=21%). mRNA expression was analyzed for genes encoding for hypoxia-inducible factor 1 alpha (*HIF1A*), hypoxia-inducible factor 2 alpha (*HIF2A*), and typical HIF target genes including glucose transporter type 1 (*SLC2A1*), vascular endothelial growth factor (*VEGF*), adrenomedullin (*ADM*), pyruvate dehydrogenase kinase 1 (*PDK1*), and the prolyl hydroxylase domain-containing proteins 1, 2 and 3 (*PHD1*, *PHD2*, *PHD3*). Expression levels of genes were normalized to beta-actin (*ACTB*) and are presented as 2-(ΔCT) values (mean±SD, N=22). Gene expression was not different during hypoxia and normoxia compared to the pre-exposure measurement, or between the two conditions (mixed-effects analysis for repeated measures or Friedman test).

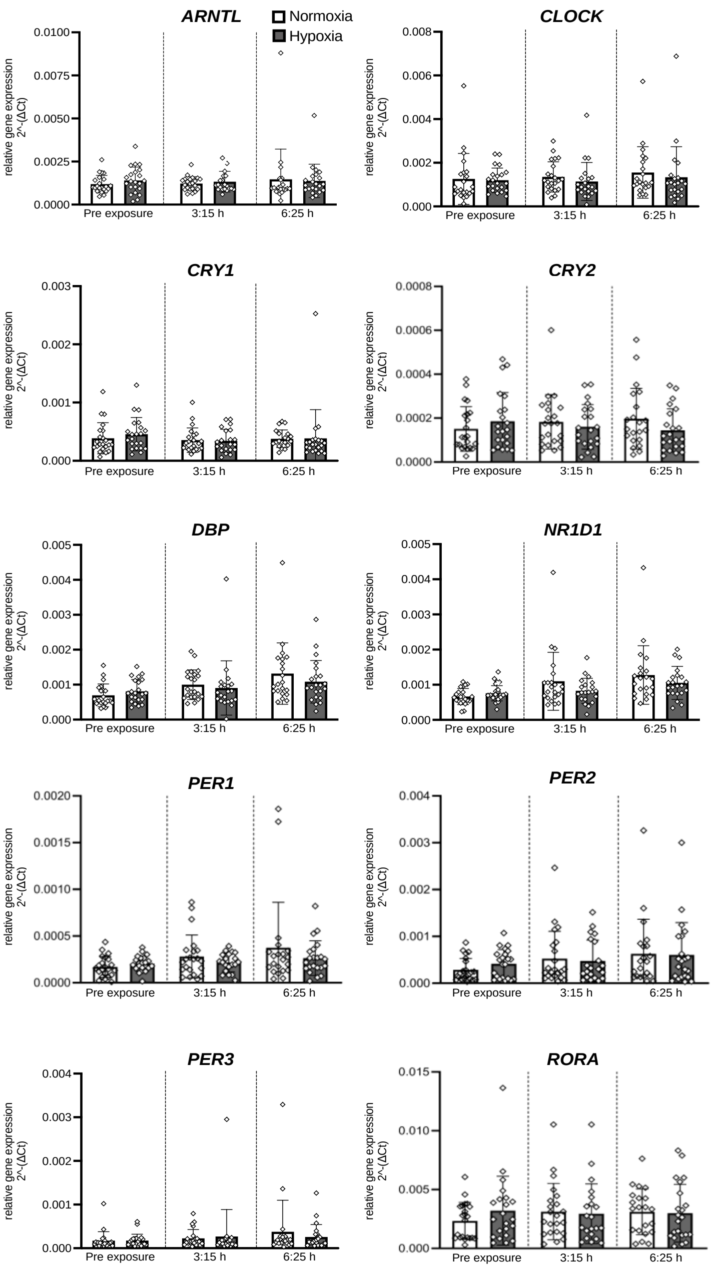

Fig. S3. Expression of clock genes in leukocytes collected from participants before and during exposure to 6.5 hours of normobaric hypoxia (FiO2=15%) and normoxia (FiO2=21%). mRNA expression was analyzed for genes encoding for aryl hydrocarbon receptor nuclear translocator-like (*ARNTL*), circadian locomotor output cycles kaput (*CLOCK*), cryptochrome circadian regulator 1 and 2 (*CRY1*, *CRY2*), albumin gene D-site binding protein (*DBP*), nuclear receptor subfamily 1, group D, member 1 (*NR1D1*), period circadian regulator 1, 2, and 3 (*PER1*, *PER2*, *PER3*), retinoic acid-related orphan receptor A (*RORA*). Expression levels of genes were normalized to beta-actin (*ACTB*) and are presented as 2-(ΔCT) values (mean±SD, N=22). Gene expression was not different during hypoxia and normoxia compared to the pre-exposure measurement, or between the two conditions (mixed-effects analysis for repeated measures or Friedman test).

Table S1. Primer sequences of specific PCR products used for RNA quantification of human whole blood cells. *ACTB*: beta-actin, *ADM*: adrenomedullin, *ARNTL*: aryl hydrocarbon receptor nuclear translocator-like, *CLOCK*: circadian locomotor output cycles kaput, *CRY1 / 2*: cryptochrome circadian regulator 1 and 2, *DBP*: albumin gene D-site binding protein, *HIF1A*: hypoxia-inducible factor 1 alpha, *HIF2A*: hypoxia-inducible factor 2 alpha, *NR1D1*: nuclear receptor subfamily 1, group D, member 1, *PER1 / 2 / 3*: period circadian regulator 1, 2 and 3, *PHD1 / 2 / 3*: pyruvate dehydrogenase kinase 1, 2 and 3, *PDK1*: pyruvate dehydrogenase kinase 1, *RORA*: retinoic acid-related orphan receptor A, *SLC2A1*: glucose transporter type 1, *VEGF*: vascular endothelial growth factor.

Gene	Primer	Sequence
ACTB	forward	CAGCGGAACCGCTCATTGCCAATGG
	reverse	TCACCCACACTGTGCCCATCTACGA
ADM	forward	AGTCGTGGGAAGAGGGAACT
	reverse	ATCCGGACTGCTGTCTTCGG
ARNTL	forward	GTGGTGCTGGCTAGAGTGTA
	reverse	TTTCAGGCGGTCAGCTTCTT
CLOCK	forward	GGCGCTCGGTTTCTCTTCTT
	reverse	GCCAGAGCCAACTCCAGAAA
CRY1	forward	TTGGAAAGGAACGAGACGCAG
	reverse	CGGTTGTCCACCATTGAGTT
CRY2	forward	TCCCAAGGCTGTTCAAGGAAT
	reverse	TGCATCCCGTTCTTTCCCAAA
DBP	forward	CGACCGCTTGATCTGGACAC
	reverse	GCTGCAAATCCTAGGAGCGA
HIF1A	forward	CTCCATTACCCACCGCTGAA
	reverse	TCACTGGGACTATTAGGCTCAGGT
HIF2A	forward	CGGAGGTGTTCTATGAGCTGG
	reverse	AGCTTGTGTGTTCGCAGGAA
NRD1	forward	TGGACTCCAACAACAACACAG
	reverse	GATGGTGGGAAGTAGGTGGG
PER1	forward	AGTCCGTCTTCTGCCGTATCA
	reverse	AGCTTCGTAACCCGAATGGAT
PER2	forward	CTTCAGCGATGCCAAGTTTGT
	reverse	CGGATTTCATTCTCGTGGCTTT
PER3	forward	GCAGGTCTATGCCAGTGTGA
	reverse	CCACCACCATTCGGTTCTGT
PHD1	forward	TGGCCCTGGACTATATCGTG
	reverse	GGCACCAATGCTTCGACAG
PHD2	forward	GCACGACACCGGGAAGTT
	reverse	CCAGCTTCCCGTTACAGT

PHD3	forward	CACAGCGAGGGAATGAACCT
	reverse	TCCTGCTGTTAAGGCTTCCG
PDK1	forward	TGAACGGATGGTGTCCTGAG
	reverse	GGCCAGGTGGACTTCTACG
RORA	forward	CACGACGACCTCAGTAACTACA
	reverse	TGGTGAACGAACAGTAGGGAA
SLC2A1	forward	CTAGCGCGATGGTCATGAGT
	reverse	TCTGGCATCAACGCTGTCTT
VEGF	forward	CCGCCTCGGCTTGTCACA
	reverse	GCAAGACAAGAAAATCCCTGTGGGCC
